# *In vivo* Pharmacokinetic and Pharmacodynamic (PK/PD) Modeling and Establishment of the PK/PD Cutoff of Florfenicol Against *Pasteurella multocida* in Ducks

**DOI:** 10.3389/fmicb.2020.616685

**Published:** 2021-01-11

**Authors:** Xia Xiao, Weixuan Lan, Yaqin Zhao, Ruichao Li, Yuan Liu, Juan Liu, Zhiqiang Wang

**Affiliations:** ^1^College of Veterinary Medicine, Yangzhou University, Yangzhou, China; ^2^Jiangsu Co-innovation Center for Prevention and Control of Important Animal Infectious Diseases and Zoonoses, Yangzhou, China; ^3^Xinjiang Institute of Chinese Materia Medica and Ethnical Materia, Wulumuqi, China; ^4^Xinjiang Key Laboratory of Chinese Materia Medica and Ethnic Materia Medica, Wulumuqi, China; ^5^Institute of Comparative Medicine, Yangzhou University, Yangzhou, China; ^6^Pizhou Animal Health Supervision Institute, Xuzhou, China; ^7^Institutes of Agricultural Science and Technology Development, Yangzhou, China

**Keywords:** florfenicol, *P. multocida*, PK/PD modeling, PK/PD cutoff, dose

## Abstract

*Pasteurella multocida* can invade and translocate through endothelial cells and result in vascular-system infection, which can cause severe economic losses in the poultry industry. Antibacterial therapy (especially florfenicol) plays an important part in controlling *P. multocida* infection. To preserve the effect of florfenicol, *in vivo* pharmacokinetic/pharmacodynamic (PK/PD) modeling of florfenicol against three *P. multocida* strains in duck was established. Then, the efficacy of the currently marketed dose, a rational dosage regimen for populations, and the PK/PD cutoff were predicted through Monte Carlo simulations (MCSs). The area under the concentration–time curve from 0 to 24 h/minimum inhibitory concentration (AUC_0–24 *h*_/MIC) was the optimal PK/PD parameter. The PK/PD surrogate values of florfenicol against *P. multocida* were similar using different organs as the PD target, but varied in different strains. For the florfenicol-sensitive strain 0825Y_1_, when the AUC_0–24 *h*_/MIC reached 117.54 and 108.19, florfenicol showed a bactericidal effect in the liver and lung, respectively. For the florfenicol-sensitive strain 0901J_1_, the corresponding value was 78.39 and 54.30, respectively. For the florfenicol-resistant strain JY160110, florfenicol could attain a maximum effect of 1 – log_10_ reduction in bacteria in the liver and lung when the AUC_0–24 *h*_/MIC reached 2.03 and 2.06, respectively. The PK/PD-based prediction for the population dose indicated a poor effect for the low end of the currently marketed dose (40 mg/kg body weight per day), but a robust effect for the high end of the currently marketed dose (60 mg/kg body weight per day) with a target attainment rate of 92.79% and 81.44% against *P. multocida* in mainland China and worldwide, respectively. The recommended dose optimized by MCSs was 52 mg/kg body weight in mainland China. The PK/PD cutoff of florfenicol against *P. multocida* at the low end and high end of the current daily dose (40 and 60 mg/kg body weight) and predicted daily dose in mainland China (52 mg/kg body weight) was 0.25, 4, and 0.5 μg/ml, respectively. These results suggested that more than one strain should be involved for PK/PD modeling and contributed to rational use of florfenicol in populations. We also provided fundamental data for determination of florfenicol breakpoints in poultry.

## Introduction

*Pasteurella multocida* is an intracellular bacterium. It can invade and translocate through endothelial cells and results in vascular-system infection in several species (e.g., cows) (Galdiero et al., 2001). *P. multocida* can infect a wide spectrum of hosts, including birds and mammals (including humans) (Wilson and Ho, 2013).

One of the most common diseases caused by *P. multocida* in animal husbandry is fowl cholera. The latter occurs sporadically or in an enzootic manner worldwide and can cause substantial economic losses. It has been reported that wild birds, mammals, and carrier pigeons may be sources of infection to commercial poultry. Investigations have indicated that carriers of *P. multocida* may exist within poultry flocks with no history of previous outbreaks of fowl cholera (Christensen and Bisgaard, 2000). Thus, confinement and vaccination are probably the most effective ways to control *P. multocida* infection.

However, extensive management systems dominant in many parts of the world and lack of efficacious live vaccines have hampered prevention of *P. multocida* infection. As a result, *P. multocida* control and treatment of fowl cholera have relied mainly on antimicrobial agents.

Florfenicol is a phenicol and has been approved to prevent and treat infectious diseases in animals (Pilehvar et al., 2016; Lan et al., 2019). Because of its strong antibacterial effect against Gram-positive and Gram-negative bacteria as well as bacteria resistant to thiamphenicol, florfenicol has an important role in the poultry industry. Despite its frequent use in livestock, resistance to florfenicol was <1% in 5356 *P. multocida* strains isolated from respiratory infections in animals (including food-producing animals and pets) between 2012 and 2017 (Bourely et al., 2019). However, with horizontal transmission of florfenicol-resistant genes such as *fexB*, *fexA*, *cfr*, and *floR*, florfenicol resistance to *Escherichia coli*, *Salmonella* species, *Riemerella anatipestifer*, and *P. multocida* is becoming increasingly serious (Zhao et al., 2016). For example, in one Taiwan district, the prevalence of resistance of *P. multocida* isolated from pigs with respiratory disease during 2013–2015 was ≤91.9%, and the minimum inhibitory concentration required to inhibit the growth of 90% of organisms (MIC_90_) was 256 μg/ml (Yeh et al., 2017). Thus, preservation of the efficacy of florfenicol is important.

It has been reported that a rational antibacterial dosage regimen based on pharmacokinetic and pharmacodynamic (PK/PD) modeling (especially *in vivo*) could maximize the therapeutic effect and minimize the emergence of resistance (Li et al., 2017; Xiao et al., 2018c). To achieve this aim, several PK/PD studies of florfenicol have been undertaken. Lei and colleagues studied the *ex vivo* PK/PD modeling of florfenicol against *Streptococcus suis* in pigs. They found that florfenicol showed a bactericidal effect when the area under the concentration–time curve from 0 to 24 h (AUC_0–24 *h*_)/MIC in serum reached 44.02 (Lei et al., 2018). Dorey and coworkers investigated the PK/PD parameter of florfenicol against pneumonia pathogens in pigs (*Actinobacillus pleuropneumoniae* and *P. multocida*) using *in vitro* PK/PD modeling. They found that florfenicol showed a bactericidal effect against *P. multocida* when the AUC_0–24 *h*_/MIC in serum reached 37.3 (Dorey et al., 2017). Sidhu and colleagues found that florfenicol showed a bactericidal effect in serum, transudates and exudates with an AUC_0–24 *h*_/MIC of 18.06, 17.34, and 17.86, respectively, using *ex vivo* PK/PD modeling in calves (Sidhu et al., 2014). PK/PD studies of florfenicol have also been undertaken in aquatic livestock (Bourely et al., 2019; Lan et al., 2019). From those studies, we can conclude that florfenicol shows good antibacterial activity against respiratory pathogens and that the best PK/PD parameter is AUC_0–24 *h*_/MIC. However, the value of AUC_0–24 *h*_/MIC to attain a certain effect differs in different animals, matrices, or using different PK/PD models. Although several PK/PD studies have focused on *P. multocida* in pigs or calves, they were conducted *in vitro* or *ex vivo*. There are differences between *in vitro* or *ex vivo* PK/PD modeling and clinical situations. Thus, investigating *in vivo* PK/PD modeling in target animal species is very important.

Antimicrobial susceptibility testing (AST) is the basis of prudent and rational use of antimicrobial agents. Clinical breakpoints are critical parameters to separate susceptible bacteria from resistant bacteria in AST. In general, it is considered that antimicrobial drugs should not be used to treat infection involving resistant bacteria. Thus, setting the value of clinical breakpoints is very important (Toutain et al., 2019). Based on guidance set by the Veterinary Committee on Antimicrobial Susceptibility Testing [a subcommittee of the European Union Committee on Susceptibility Testing (EUCAST)], clinical breakpoints were determined according to the relationship between the epidemiological cutoff, PK/PD cutoff, and clinical cutoff (Toutain et al., 2017; Turnidge and Martinez, 2017). The PK/PD cutoff is defined as the highest possible MIC for which a given percentage of target animals (usually 90%) achieve a defined PK/PD-parameter value (e.g., PK/PD-parameter value for a bactericidal effect) (European Medicines Agency, 2015). As a result, the PK/PD-parameter value for a certain effect is crucial for determination of the PK/PD cutoff. The PK/PD cutoff is pivotal for determination of a clinical breakpoint because it reflects the relationship between the exposure and efficacy of a drug.

Monte Carlo simulations (MCSs) are mathematical methods employed to ascertain the probability of an outcome through repeated random sampling. They have been used in several fields in science and economics for data analyses and theory confirmation. MCSs are also recommended for analyses of attainment of PK/PD targets to assess antibacterial dosing regimens and determination of PK/PD cutoffs.

We established an *in vivo* PK/PD model of florfenicol in ducks against several *P. multocida* strains. To ascertain if isolates with different susceptibilities impacted PK/PD-parameter values, three *P. multocida* strains of different susceptibilities were used in PK/PD modeling. To recommend a rational dosage regimen and establish a PK/PD cutoff of florfenicol against *P. multocida*, MCSs were conducted based on the PK of florfenicol in infected ducks. The PK/PD thresholds obtained in our study and MIC distribution were derived from the literature.

## Materials and Methods

### Ethical Approval of the Study Protocol

Animal studies were approved (SYXKSU-2007-0005) by the Jiangsu Administrative Committee for Laboratory Animals (Jiangsu, China). All procedures complied with the guidelines for laboratory animal welfare and ethics set by the Jiangsu Administrative Committee for Laboratory Animals (Jiangsu, China). All experiments involving live bacterium and animals were conducted in biosafety level 2 animal facilities in accordance with the institutional bio-safety manual.

### Chemicals

Florfenicol standard (CAS: 73231-34-2; purity, ≥99%; lot no. Y05J6C1) was purchased from Yuanye Biological Technology (Shanghai, China). Florfenicol as a raw material (purity, ≥95%; O1004A) and other antimicrobial agents were supplied by Xiangbo Biological Technology (Guangzhou, China).

### Bacteria

Strain C_48–1_ is a standard type of strain of *P. multocida* and was kindly provided by Jiangsu Institute of Poultry Sciences (Jiangsu, China). The other 11 strains were isolated from ducks diagnosed with fowl cholera and kindly supplied by the clinical veterinary laboratory at Yangzhou University (Yangzhou, China). Strains were cultured using Mueller Hinton agar or Mueller Hinton broth (MHB) containing 5% calf serum.

### Animals

Gaoyou ducks (6 weeks, 0.7 ± 0.1 kg) were provided by Gaoyou Suyou Duck Farm (Yangzhou, China). Animals were housed under standard conditions and fed with antibiotic-free balanced feed and water. Animals were allowed to acclimatize to their environment for 1 week before experimentation.

### *In vitro* PD Study

The MIC of florfenicol, cefquinome, gentamicin, tilmicosin, and enrofloxacin against 12 strains of *P. multocida* was evaluated using the microdilution method according to guidelines set by the Clinical and Laboratory Standards Institute (CLSI, Beijing, China). The MIC of florfenicol in duck serum against the 12 strains was also determined using the microdilution method with a matrix of duck serum. The minimum bactericidal concentration (MBC) and mutant prevention concentration (MPC) of florfenicol were determined as described in our previous study (Xiao et al., 2018a). The *in vitro* time-killing curve of florfenicol against three strains with different initial bacteria concentrations was also studied in artificial medium.

### Model of *P. multocida* Infection

A model of *P. multocida* infection was established according to our previous study with three isolates of different susceptibility (Lan et al., 2019). Briefly, strains 0825Y_1_ (sensitive to all five antimicrobial agents), 0901J_1_ (sensitive to florfenicol, but resistant to gentamicin and enrofloxacin), and JY160110 (resistant to all antimicrobial agents except gentamicin) were grown in broth overnight.

Then, they were centrifuged and resuspended in physiologic (0.9%) saline. For each isolate, 30 ducks were divided into six groups of five. Ducks were administered 0.2 ml of 0.9% saline containing 0, 10^2^, 10^3^, 10^4^, 10^5^, or 10^6^ colony-forming units (CFU)/ml of bacteria through tracheal instillation. Twelve hours later, the clinical symptoms, anatomy, and pathological changes were monitored. Next, animals were sacrificed by lethal injection of Beuthanasia solution (0.3 ml/kg, intravenously) after anesthesia with ketamine–xylazine. Light microscopy and real-time reverse transcription–quantitative polymerase chain reaction (RT-qPCR) were used for bacteria identification with the primer of *kmtl* (forward: 5′-ATCCGCTATTTACCCAGTGG-3′; reverse: 5′-GCTGTAAACGAACTCGCCAC-3′). The bacterial load in the liver and lung was estimated via dilution of tissue homogenates and plating onto MHB plate. Bacterial colonies were counted after incubation for 16–18 h at 37°C. The mortality rate was monitored until 84 h after infection.

### PK of Florfenicol in Infected Ducks

Forty-eight infected ducks were fasted for 12 h and divided randomly into four groups with equal numbers of males and females in each group. Each group was treated via the intragastric route with a single dose of 1, 15, 30, or 60 mg/kg body weight. Blood samples were collected at 0, 0.167, 0.5, 1, 1.5, 2, 4, 8, 12, and 24 h. Then, samples were incubated at 37 and 4°C for 1 and 2 h, respectively. Then, serum was separated by centrifugation at 3000 × *g* for 10 min at room temperature.

The florfenicol concentration in serum was determined using high-performance liquid chromatography (HPLC) as we reported previously (Lan et al., 2019). Briefly, florfenicol was extracted with 2 ml of ethyl acetate, dried in a nitrogen stream, and dissolved in 0.2 ml of mobile phase [water and acetonitrile at 75:25 (vol:vol)]. Florfenicol was separated with a Hypersil ODS2 C18 column (250 mm × 4.6 mm, 5 μm; Yilite, Dalian, China) and underwent UV detection at 225 nm in a 1260 HPLC system (Agilent Technologies, Santa Clara, CA, United States). The recovery rate was 82.43%–97.17%. The intraday and interday coefficient of variation was 5%, respectively. The limit of quantification (LoQ) was 0.05 μg/ml. PK analyses were undertaken using a noncompartmental model provided in WinNonlin 6.1 (Pharsight, Mountain View, CA, United States). The relationship between AUC_0–12 *h*_ and dose was simulated with Excel^TM^ (Microsoft, Redmond, WA, United States).

### *In vivo* PD Study of Florfenicol

A total of 135 Gaoyou ducks (6 weeks) were divided into three groups. They were infected with 0825Y_1_, 0901J_1_, or JY160110 strains, respectively. In each group, animals were divided into nine subgroups. Florfenicol was administrated (p.o.) to each subgroup at 0, 1, 2, 5, 8, 10, 15, 20, or 30 mg/kg body weight twice daily for three successive days. After the final administration, ducks were sacrificed. One side of the lung and liver was collected to calculate the burden of *P. multocida* in these organs. Changes of bacterial abundance in each organ were calculated.

### PK and PD Analyses

It has been reported that the best PK/PD parameter for florfenicol is the AUC/MIC. Therefore, the AUC_0–24 *h*_/MIC was calculated according to PK data and *in vitro* PD data. Changes in bacterial abundance in organs were integrated with the PK/PD parameters AUC_0–24 *h*_/MIC using the sigmoid *E*_*max*_ model with WinNonlin using Eq. 1. The PK/PD parameters required for bacteriostasis and a bactericidal effect were also calculated.

(1) $$E=E_{0}-\frac{E_{\text{max}}\times C^{N}}{C^{N}+{\text{EC}}_{50}^{N}}$$

where *E*_0_ is the change in log_10_ CFU/organ in the control sample (absence of florfenicol); *E*_*max*_ is the difference in effect between the greatest amount of growth (as seen for the growth control, *E*_0_) and the greatest amount of killing; *C* is the AUC_0–24 *h*_/MIC in the affected compartment; EC_50_ is the AUC_0–24 *h*_/MIC producing a 50% reduction in the bacterial count; and *N* is the Hill coefficient that describes the steepness of the AUC_0–24 *h*_/MIC-effect curve (Burton, 2005).

### Prediction of the Population Dose and Establishment of PK/PD Cutoff by MCS

A 10,000-subject MCS was conducted using Crystal Ball Professional V7.2.2 (Oracle, Redwood City, CA, United States) based on the PK results for florfenicol in infected ducks in the present study; indices of PK/PD targets obtained in the present study; MIC distribution of florfenicol against 4314 *P. multocida* from 1987 to 2015 in different world regions collected from the National Center for Biotechnology Information database (Lizarazo et al., 2006; Tang et al., 2009; Portis et al., 2012; Dayao et al., 2014; de Jong et al., 2014; El Garch et al., 2016; Anholt et al., 2017; Yeh et al., 2017). The PK parameter AUC_0–24 *h*_ was assumed to have a log-normal distribution in the form of mean values and confidence intervals. Under different MIC distributions, distribution of the AUC_0–24 *h*_/MIC was calculated through MCS. The target attainment rate (TAR) at the existing daily dose was evaluated under the MIC distribution in mainland China, Taiwan (which is part of China), and worldwide. Rational doses were recommended with the principle of TAR reaching 50% TAR, 90% TAR (or 85% TAR) in mainland China, Taiwan, and worldwide. The PK/PD cutoff was the MIC, at which the TAR for a 3-log, 10-unit decrease equaled 90% under the recommended dose.

## Results

### *In vitro* PD

**FIGURE 1:**
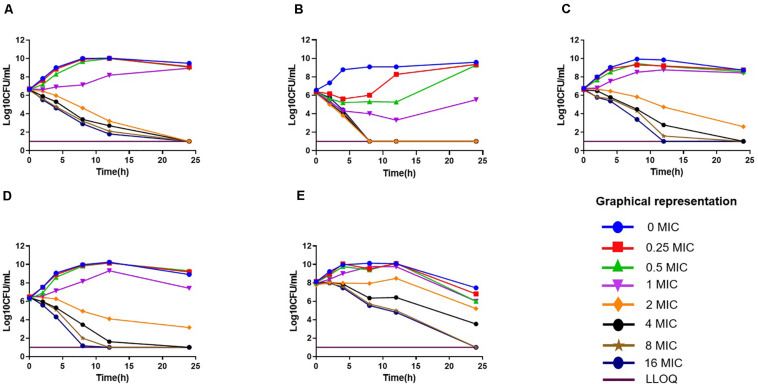
*In vitro* time-killing curves of florfenicol against *Pasteurella multocida*. **(A)** C_48–1_; **(B)** JY160110; **(C)** 0825Y_1_; **(D)** 0901J1; **(E)** 0825Y1.

The *in vitro* antimicrobial effect of drugs against 12 isolates is shown in Supplementary Table S1. The MIC range of florfenicol against 12 strains of *P. multocida* was 0.125–32 μg/ml in broth, which was slightly lower than that in duck serum (0.25–32 μg/ml). The MIC range (in μg/ml) of cefquinome, gentamicin, tilmicosin, and enrofloxacin was 0.25–128, 4–8, 1–128, and 0.25–2, respectively. The strains 0825Y_1_ (sensitive to all five antimicrobial agents), 0901J_1_ (sensitive to florfenicol, but resistant to gentamicin and enrofloxacin), and JY160110 (resistant to all antimicrobial agents except gentamicin) were chosen for further study. The MBC of florfenicol was 2× MIC and ranged from 0.25 to 64 μg/ml. The MPC of florfenicol was 1.6–2× MIC. *In vitro* time-killing curves showed that, if the initial bacterial concentration was 10^6^ CFU/ml, 2× MIC reached a bactericidal effect, whereas the minimum value was 4× MIC if the initial bacterial concentration was 10^8^ CFU/ml (Figure 1).

### Model of *P. multocida* Infection

Neither clinical signs nor anatomopathological lesions were observed in ducks of the control group. In accordance with our previous report, ducks inoculated with 10^3^–10^6^ CFU of each isolate died within 24 h, whereas ducks given 10^2^ CFU survived >36 h. Clinical symptoms (e.g., tachypnea, intolerance of cold, depression, decreased feeding, diarrhea) were observed in all infected groups. Anatomical lesions such as edema and hemorrhage in the stomach and intestines, fluid accumulation in the pericardium, and small gray necrotic spots in the liver and spleen were observed (Supplementary Figure S1). The symptoms worsened as the bacterial inoculation increased. The RT-qPCR result is shown in Supplementary Figure S2. Identical bands were observed for all samples infected with the three isolates. Therefore, the scheme of the infection model was tracheal inoculation with 200 μl of a bacterial suspension of ∼10^2^ CFU/ml. Twelve hours after inoculation, the bacterial burden was about 10^6^–10^7^ CFU in the liver and lung (Table 1).

**TABLE 1:** The bacterial burden of the liver and lung in ducks after 12-hours infection with 10^2^ CFU/ml *Pasteurella multocida* (200 μl) (mean ± SD, *n* = 5).

Strain	Log_10_ CFU/liver	Log_10_ CFU/lung
0825Y_1_	6.07 ± 0.56	6.00 ± 0.71
0901J_1_	6.12 ± 0.33	7.37 ± 0.68
JY160110	6.63 ± 0.79	6.94 ± 0.48

### Pharmacokinetics

**FIGURE 2:**
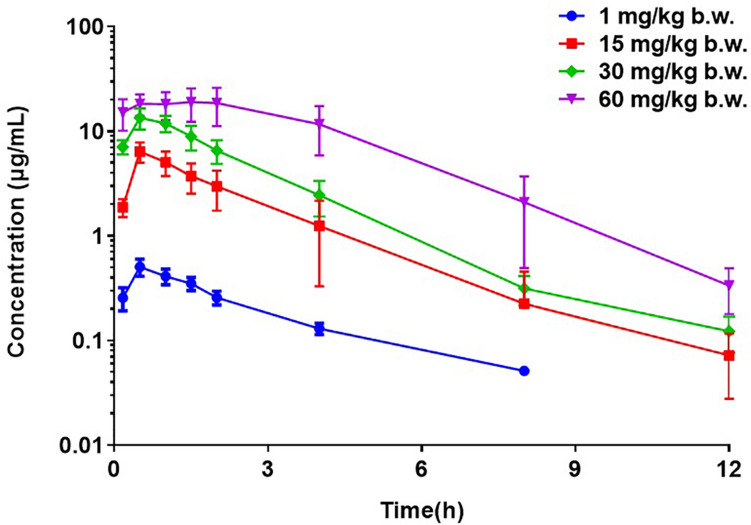
Semi-concentration–time profile of florfenicol in serum after oral administration at doses of 1, 15, 30, and 60 mg/kg body weight in *P. multocida*-infected ducks (*n* = 12).

The PK profiles of florfenicol and the main PK parameters are presented in Figure 2 and Table 2, respectively. The serum concentration of florfenicol for all doses was lower than the LoQ at 24 h. For a dose of 1 mg/kg body weight, florfenicol was undetectable at 12 h. The time for peak concentration (*T*_*max*_) and elimination half-life (*T*_½β_) for all four doses showed little difference. However, the AUC_0–24 *h*_ increased as the dose increased. A good linear relationship between the dose and AUC_0–24 *h*_ in the range 1–60 mg/kg body weight was observed (*r*^2^ = 0.9734). Thus, the AUC_0–24 *h*_ for doses 1–60 mg/kg body weight could be deduced according to this linear relationship. For doses of 2, 5, 8, 10, and 20 mg/kg body weight, the AUC_0–24 *h*_ was 4.58, 11.93, 18.19, 22.73, and 45.41 respectively.

**TABLE 2:** Serum pharmacokinetic parameters of florfenicol in *P. multocida*-infected ducks following oral administration at doses of 1, 15, 30, and 60 mg/kg body weight (*n* = 12).

Dose (mg/kg body weight)	*C*_*max*_ (μg/ml)	*T*_*max*_ (h)	AUC_0–12 *h*_ (μg h/ml)	*T*_1/2__β_ (h)
1	0.51 ± 0.10	0.54 ± 0.14	1.66 ± 0.18	1.83 ± 0.27
15	6.52 ± 1.37	0.67 ± 0.32	16.05 ± 6.38	1.83 ± 0.21
30	13.88 ± 2.70	0.67 ± 0.25	34.52 ± 8.29	1.62 ± 0.07
60	22.35 ± 6.56	0.97 ± 1.16	97.24 ± 37.95	1.61 ± 0.18

### *In vivo* PD of Florfenicol Against *P. multocida*

**FIGURE 3:**
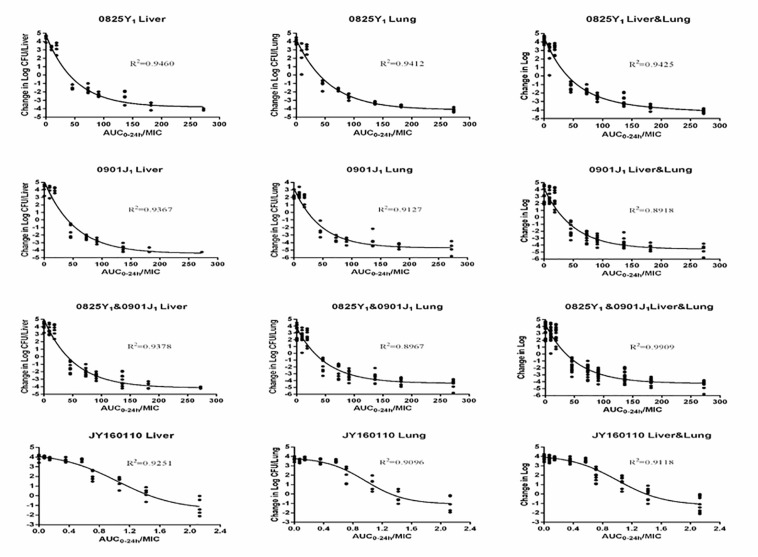
Sigmoid *E*_*max*_ relationships between antibacterial effect (log_10_ CFU) in the liver or lung and AUC_0–24 *h*_/MIC against *P. multocida* in ducks.

Ducks administered 0.9% saline or a low dose of florfenicol showed extensive symptoms of fowl cholera. The bacterial load was as high as 10-log_10_/organ. However, as the florfenicol dose increased, the bacterial load decreased. The profiles of the sigmoid *E*_*max*_ model describing the relationship between the AUC_0–24 *h*_/MIC and antibacterial efficacy in each organ are presented in Figure 3. The AUC_0–24 *h*_/MIC was the optimal PK/PD parameter, with a correlation coefficient of ∼0.9 for all three strains. For the sensitive strain 0825Y_1_, when the AUC_0–24 *h*_/MIC reached 117.54 and 108.19, florfenicol showed a bactericidal effect in the liver and lung, respectively. For the florfenicol-sensitive but gentamicin-intermediary and enrofloxacin-resistant strain 0901J_1_, the AUC_0–24 *h*_/MIC was 78.39 and 54.30, respectively. However, for the florfenicol-resistant strain JY160110, florfenicol could not attain a bactericidal effect at the highest dose. When the AUC_0–24 *h*_/MIC reached 2.03 and 2.06, it showed a 1 – log_10_ killing effect in the liver and lung, respectively (Table 3). The PK/PD-parameter values of florfenicol against *P. multocida* showed little difference using different organs as the PD target, but varied in different strains.

**TABLE 3:** *In vivo* PK/PD parameters of florfenicol against *P. multocida* based on the targets of the liver and lung.

Strain	Parameter	Liver	Lung	Liver and lung
0825Y_1_	*E*_*max*_ (log_10_ CFU/liver or lung)	8.30	8.04	8.21
	*E*_0_ (log_10_ CFU/liver or lung)	4.40	3.80	4.12
	EC_50_	36.70	42.47	39.57
	AUC_0–24 *h*_/MIC for bacteriostatic	39.23	39.99	39.73
	AUC_0–24 *h*_/MIC for bactericidal	117.54	108.19	113.57
	Slope (N)	1.81	1.82	1.78
0901J_1_	*E*_*max*_ (log_10_ CFU/liver or lung)	8.23	6.80	7.57
	*E*_0_ (log_10_ CFU/liver or lung)	4.33	2.50	3.42
	EC_50_	37.94	33.63	35.94
	AUC_0–24 *h*_/MIC for bacteriostatic	39.34	28.09	33.66
	AUC_0–24 *h*_/MIC for bactericidal	78.39	54.30	64.38
	Slope (N)	2.89	3.01	2.95
0825Y_1_ and 0901J_1_	*E*_*max*_ (log_10_ CFU/liver or lung)	8.12	7.31	7.69
	*E*_0_ (log_10_ CFU/liver or lung)	4.31	3.14	3.71
	EC_50_	37.17	37.32	37.27
	AUC_0–24 *h*_/MIC for bacteriostatic	39.16	33.01	36.18
	AUC_0–24 *h*_/MIC for bactericidal	94.04	76.49	83.92
	Slope (N)	2.37	2.31	2.37
JY160110	*E*_*max*_ (log_10_ CFU/liver or lung)	6.43	5.11	5.57
	*E*_0_ (log_10_ CFU/liver or lung)	3.98	3.68	3.83
	EC_50_	1.23	1.00	1.08
	AUC_0–24 *h*_/MIC for bacteriostatic	1.50	1.33	1.42
	AUC_0–24 *h*_/MIC for 1 – log_10_ reduction	2.03	2.06	2.06
	Slope (N)	2.46	3.31	2.90

### Prediction of the Population Dose and Establishment of the PK/PD Cutoff by MCS

**FIGURE 4:**
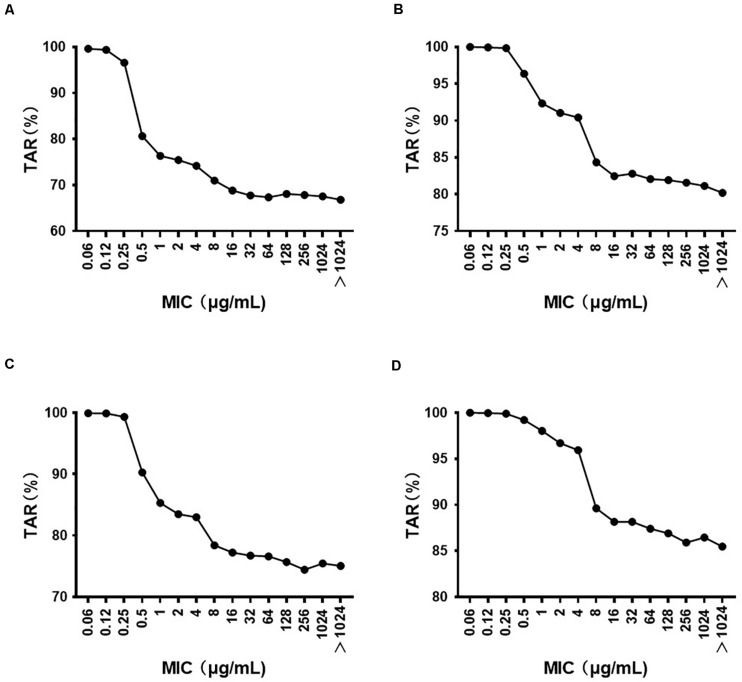
The target attainment rate (TAR) of florfenicol against *P. multocida* under different MIC distributions at a daily dose (in mg/kg body weight) of **(A)** 40; **(B)** 60; **(C)** 52; **(D)** 90.

The MIC distribution of florfenicol against 4314 *P. multocida* derived from the literature is summarized in Supplementary Table S2. The MIC distribution in mainland China, Taiwan, and worldwide is shown in Supplementary Figure S3. The MIC_50_ and MIC_90_ (in μg/ml) in mainland China and Taiwan were 0.25 and 0.5, and 64, 256, respectively, which represented florfenicol-sensitive and florfenicol-resistant areas. Thus, when conducting MCSs, the PK/PD surrogate value attained a certain effect derived from florfenicol-sensitive strains (0825Y_1_, 0901J_1_) used in mainland China, whereas the value derived from the florfenicol-resistant strain JY160110 was used for Taiwan. The TAR of florfenicol at the high end of the existing daily dose (60 mg/kg body weight per day) against *P. multocida* under MIC distribution in mainland China, Taiwan, and worldwide is shown in Supplementary Figures S4–S6 and summarized in Table 4. The TAR was 85.98% and 66.84% in mainland China and worldwide, respectively, and was 30.09% in Taiwan. In mainland China, the daily dose (in mg/kg body weight) needed to achieve 50% TAR and 90% TAR was 21 and 52, respectively; worldwide, the daily dose needed to achieve 50% TAR and 85% TAR was 33 and 90, respectively; in Taiwan, the daily dose needed to achieve 50% TAR was 84. The cutoff values of florfenicol against *P. multocida* at the existing daily dose (40 and 60 mg/kg body weight) and predicted daily doses (52 and 90 mg/kg body weight) were 0.25, 4, 0.5, and 4 μg/ml, respectively (Figure 4).

**TABLE 4:** The target attainment rate (TAR) of florfenicol at the high end of the existing dose (60 mg/kg body weight per day) under different MIC distributions (%).

Target		Mainland China	Worldwide	Taiwan
0825Y_1_	Liver	85.98	66.84	
	Lung	88.52	72.94	
	Liver and lung	87.00	68.86	
0901J_1_	Liver	93.49	82.58	
	Lung	96.29	86.59	
	Liver and lung	95.33	84.81	
0825Y_1_ and 0901J_1_	Liver	91.23	77.61	
	Lung	93.88	83.05	
	Liver and lung	92.79	81.44	
JY160110	Liver			31.78
	Lung			30.09
	Liver and lung			30.86

## Discussion

*Pasteurella multocida* infection causes severe economic losses in the poultry industry. Antibacterial therapy (especially florfenicol) plays an important part in controlling *P. multocida* infection.

To preserve the effect of florfenicol against *P. multocida*, an *in vivo* PK/PD model of florfenicol against *P. multocida* in ducks using isolates of different sensitivity was established. Based on the florfenicol PK in infected ducks, the PK/PD thresholds obtained and MIC distribution, and the adequacy of the current florfenicol dose, a rational population dose regimen and PK/PD cutoff were predicted through MCSs.

The MIC of florfenicol against 12 *P. multocida* isolates in artificial medium and duck serum showed only a slight difference. Similar results have been found for the MIC of florfenicol against *P. multocida*, *A. pleuropneumoniae*, and *Mannheimia haemolytica* in the sera of calves and pigs (Sidhu et al., 2014; Dorey et al., 2017). Hence, the protein binding rate of florfenicol in animal sera is low. MBC/MIC and MPC/MIC were 2 and 1.6–2, respectively, which was in accordance with previous reports. MBC/MIC and MPC/MIC of florfenicol against *Streptococcus* species were 2 and 1.6, respectively (Sidhu et al., 2014; Lei et al., 2018). The low MPC/MIC indicated that the mutant selection window (MSW) of florfenicol was narrow, and it was possible to close the MSW of florfenicol through an appropriate dosing regimen. With identical initial bacteria concentrations, the concentration needed for a bactericidal effect increased. The initial bacteria concentration is crucial for time-killing curves. Identical conclusions have also been found in work by Xiao et al. (2018c).

Creation of a disease model is very important for *in vivo* PK/PD modeling. In the present study, to mimic the clinical situation, the immunity of animals was not destroyed. This strategy hampered establishment of a disease model with similar bacterial loads in the lung and liver among different animals. Several doses (10^2^–10^6^ CFU) and administration methods (intraperitoneal injection, intramuscular injection, tracheal instillation) were attempted. Finally, we found that when the target organ, lung, was infected directly through tracheal instillation at ∼10^2^ CFU, the bacterial loads in lung and liver among different animals were similar for all three test strains. This dose was similar to that used in duck infection through intramuscular injection (∼10^2^ CFU) (Xiao et al., 2018b), but much lower than that used in mice (10^8^ CFU) and rabbits (10^6^ CFU) for lung infection with *P. multocida* (Elazab et al., 2018; Zeng et al., 2018). One reason could be that ducks are more susceptible to *P. multocida* than mammals and that the isolate used in mice and rabbits (CVCC1669; serotype, B:2) is not a predominant serotype for infection in mammals.

Three strains of different sensitivity were used in our *in vivo* PD study. The bacteria reduction in the lung (BRLG) and liver (BRLR) was calculated using PD surrogates and thereafter. For a particular strain, the threshold for the AUC_0–24 *h*_/MIC for a certain effect was similar using BRLG or BRLR as the PD surrogate. However, for fluoroquinolones, opposite results have been illustrated. The AUC_0–24 *h*_/MIC for danofloxacin against *Salmonella typhimurium* for a bactericidal effect has been reported to be 121.30, 354.28, 216.64, and 228.66 in the blood, liver, spleen, and lung, respectively (Xiao et al., 2018c). The AUC_0–24 *h*_/MIC for enrofloxacin to produce a bactericidal effect against *E.coli* has been reported to be 21.29, 41.68, and 27.65 in blood, liver, and lung, respectively (Xiao et al., 2018a). The reason for this discrepancy may be that the distribution of fluoroquinolones in different organs varies, whereas the concentration of florfenicol in the liver and lung is similar. However, the threshold of the AUC_0–24 *h*_/MIC for a certain effect was very different between different isolates using BRLG or BRLR as the PD surrogate. Although the MIC of florfenicol against 0825Y_1_ and 0901J_1_ was identical in medium and serum, the AUC_0–24 *h*_/MIC for a bactericidal effect in the lung was 108.19 and 54.30, respectively. The value for 0825Y_1_ was more than double that for 0901J_1_.

A similar phenomenon has been observed in PK/PD modeling of colistin against *Pseudomonas aeruginosa*. The AUC_*ELF*_/MIC of colistin for a bacteriostatic effect in the lung against ATCC27853, PAO1, and FADDI-PAO22 strains were 1050, 971, and 684, respectively, although their MIC was identical. The maximum effect of colistin was 1 – log_10_ reduction against PAO1 and FADDI-PAO22 strains, but was bacteriostatic (0 - log_10_ reduction) against the ATCC27853 strain (Lin et al., 2017).

A similar action has been seen in PK/PD modeling of GSK1322322 against *Streptococcus pneumoniae*, *Haemophilus influenzae*, and *Staphylococcus aureus* (Hoover et al., 2016). The bacteria reduction was not only the result of drug action but also the action of the host immune system. There may be a discrepancy in the virulence of different bacterial strains, which would impact the interaction between host and bacteria and finally influence the drug effect. Thus, when PK/PD modeling is established, more than one strain should be involved.

The AUC_0–24 *h*_/MIC thresholds for a bactericidal effect of florfenicol against susceptible isolates were much higher than those reported by Dorey et al. (2017) and Sidhu et al. (2014). Although the AUC_0–24 *h*_ in serum/MIC correlated well with efficacy in each organ, it was the concentration in the target organ that influenced bacteria reduction. Also, tissue PK may differ in different animal species, which would impact the AUC_0–24 *h*_/MIC threshold. Moreover, in studies by Dorey and colleagues and Sidhu and coworkers, *ex vivo* PK/PD modeling was employed. We have reported the discrepancy of the AUC_0–24 *h*_/MIC threshold resulting from *ex vivo* and *in vivo* PK/PD modeling (Xiao et al., 2015, 2018a, c), and we illustrated the reasons. These results further confirmed that *in vivo* PK/PD modeling has great advantages compared with *ex vivo* PK/PD modeling and is more suitable to determine predictors of antibacterial efficacy. The AUC_0–24 *h*_/MIC threshold for a bactericidal effect obtained in the present study was higher than that against *A. pleuropneumoniae* (58.40), *M. haemolytica* (26.63), and *Streptococcus* species (44.02) (Sidhu et al., 2014; Dorey et al., 2017; Lei et al., 2018). The threshold of florfenicol against those three bacterial species was calculated through *in vitro* and *ex vivo* PK/PD modeling, which would explain (at least in part) the low threshold values. However, whether there are discrepancies of florfenicol PK/PD thresholds against different types of bacteria merits further study. It is known that antibiotics do not work against resistant bacteria, but in the present study, a 1 – log_10_ reduction was observed in resistant *P. multocida* with an MIC of 32 μg/ml after florfenicol treatment. These data suggest that florfenicol could also be used during infection with resistant bacteria if drug availability is limited.

With regard to the high end of the current florfenicol dose, the TAR was 85.98% and 66.84% in mainland China and worldwide using the PK/PD surrogate value to attain a bactericidal effect derived from florfenicol-sensitive strains (0825Y_1_, 0901J_1_); it was ∼30% in Taiwan using the value derived from the florfenicol-resistant strain JY160110. These data suggest that the existing daily dose achieved a moderate therapeutic outcome as a whole, an excellent outcome in mainland China, but a poor outcome in Taiwan. These results suggest that the current dosage regimen needs optimization in some parts of the world. Thus, we recommend a more rational dose for populations based on MIC data, the results of *in vivo* PK/PD modeling, and PK of florfenicol in infected ducks.

In mainland China, the daily dose needed to achieve 90% TAR was 52 mg/kg body weight. The dose needed to achieve 90% TAR was included in the existing daily dose (40–60 mg/kg body weight) but was higher than that recommended by Lei et al. (2018) against *S. suis* (25.02 mg/kg body weight). Worldwide, the daily dose needed to achieve 85% TAR was 90 mg/kg body weight, but because the MIC distribution varies greatly in different areas, the actual importance of these recommended doses is limited. In Taiwan, florfenicol could barely attain a bactericidal effect, and the daily dose needed to reach 50% TAR for a 1 – log_10_ reduction in bacteria was 84 mg/kg body weight, which is higher than the existing daily dose. However, there was a limitation of the recommended dose because *P. multocida* isolated from calves and pigs were used in MCSs. If available, the MIC value of *P. multocida* isolated from poultry should be used.

Clinical breakpoints are determined according to the relationship between the epidemiological cutoff, PK/PD cutoff, and clinical cutoff. The PK/PD cutoff is pivotal for determination of the clinical breakpoint because it reflects the relationship between the exposure and efficacy of a drug. The PK/PD cutoffs of florfenicol against *P. multocida* at the low end of the existing daily dose (40 mg/kg body weight) and predicted daily dose in mainland China (52 mg/kg body weight) were 0.25 and 0.5 μg/ml, respectively. Toutain et al. (2019) stated that the PK/PD cutoff of florfenicol for pathogens in calves was 1 mg/l at a dose of 40 mg/kg body weight. The drug used in their study was a long-acting formulation, whereas we used a conventional formulation. The formulation can impact the PK/PD cutoff through changing PK characteristics. These values are much lower than the susceptibility breakpoint defined by the CLSI (the susceptibility, intermediate, and resistance breakpoints of florfenicol against *P. multocida* were 2, 4, and 8 μg/ml, respectively) (Clinical and Laboratory Standards Institute [CLSI], 2018). They are also lower than the epidemiological cutoff of florfenicol against *P. multocida* (1 μg/ml) defined by EUCAST. However, the PK/PD cutoff of florfenicol against *P. multocida* at the high end of the existing daily dose (60 mg/kg body weight) was 4 μg/ml, which is higher than that defined by the CLSI and EUCAST. These results suggest that the dose range has a crucial role in determination of the PK/PD cutoff. The values defined by CLSI and EUCAST are aimed mainly at *P. multocida* isolated in calves and pigs, whereas the value in our study is aimed at *P. multocida* isolated from poultry. The target animal may also impact the results of the PK/PD cutoff.

## Conclusion

We established *in vivo* PK/PD modeling of florfenicol against *P. multocida* in ducks using isolates of different sensitivity. The AUC_0–24 *h*_/MIC was the optimal PK/PD parameter. The PK/PD surrogate values of florfenicol against *P. multocida* were similar using different organs as targets, but varied in different strains, thereby suggesting that more than one strain should be involved for PK/PD modeling in the future. The PK/PD-based dose prediction for populations indicated a poor effect for the low end of the current marketed dose (40 mg/kg body weight per day), but a robust effect for the high end of the current marketed dose (60 mg/kg body weight per day). The PK/PD cutoff of florfenicol against *P. multocida* at the low end and high end of the existing daily dose (40 and 60 mg/kg body weight) and predicted daily dose in mainland China (52 mg/kg body weight) were 0.25, 4, and 0.5 μg/ml, respectively. Our study preserved the effect of florfenicol and contributed to rational use of florfenicol in populations. It also provides fundamental data for breakpoint determination for florfenicol in poultry.

## Data Availability Statement

The original contributions presented in the study are included in the article/Supplementary Material, further inquiries can be directed to the corresponding author/s.

## Ethics Statement

The animal study was reviewed and approved by the animal studies were approved by the Jiangsu Administrative Committee for Laboratory Animals (Permission Number: SYXKSU-2007-0005).

## Author Contributions

XX and ZW designed this study and revised and guided the experiment. XX wrote this manuscript and participated in the whole experiment process. WL managed the whole experiment. YZ and JL helped with the sampling process and concentration detection. RL and YL supported for the data analysis. All authors contributed to the article and approved the submitted version.

## Conflict of Interest

The authors declare that the research was conducted in the absence of any commercial or financial relationships that could be construed as a potential conflict of interest.
