# Supplementary figures and images for: In vivo Pharmacokinetic and Pharmacodynamic (PK/PD) Modeling and Establishment of the PK/PD Cutoff of Florfenicol Against Pasteurella multocida in Ducks
Source: Front Microbiol. 2021 Jan 11;11:616685. doi: 10.3389/fmicb.2020.616685 (PMC7829356; doi:10.3389/fmicb.2020.616685)

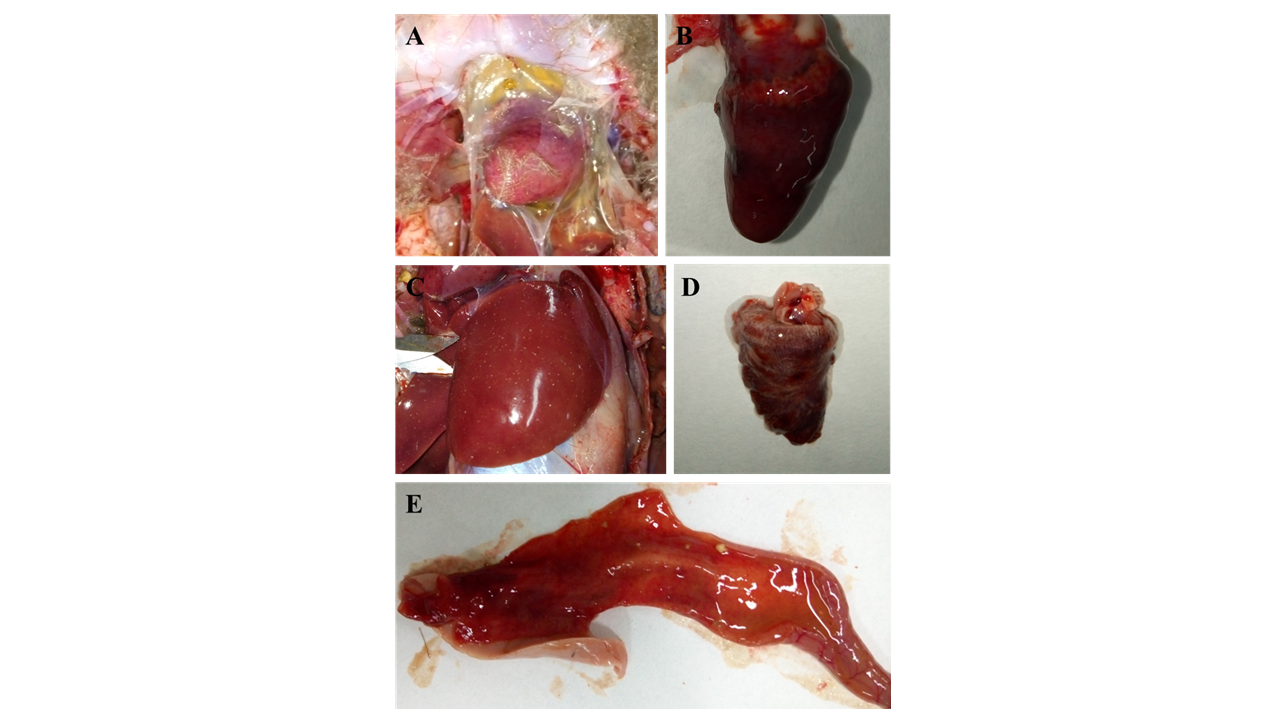

Supplement: Supplementary file 1 [file Image_1.TIF]

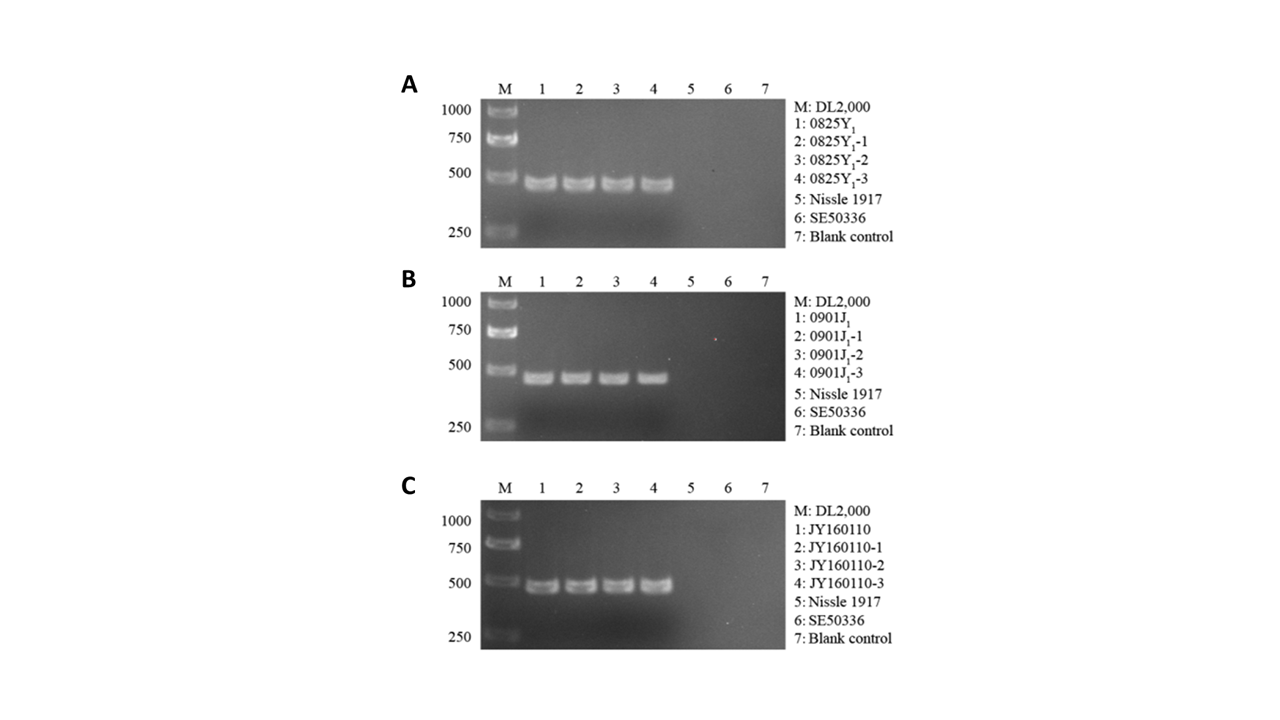

Supplement: Supplementary file 2 [file Image_2.TIF]

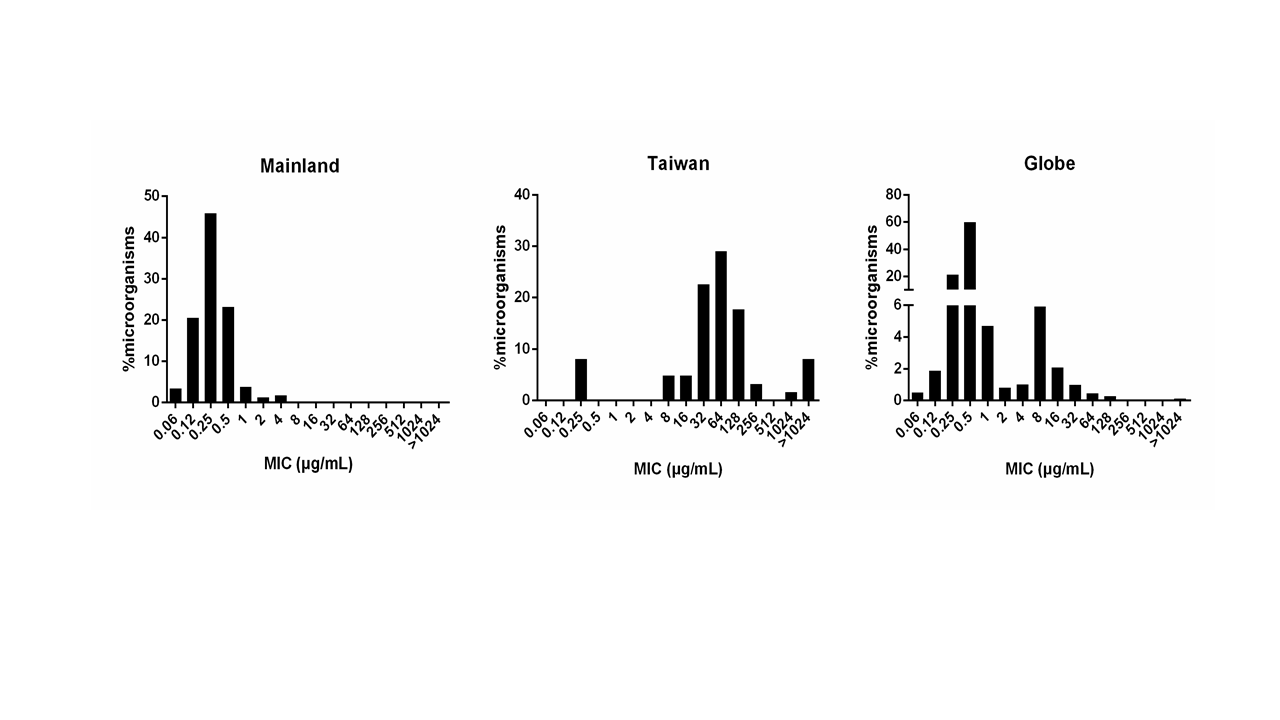

Supplement: Supplementary file 3 [file Image_3.TIF]

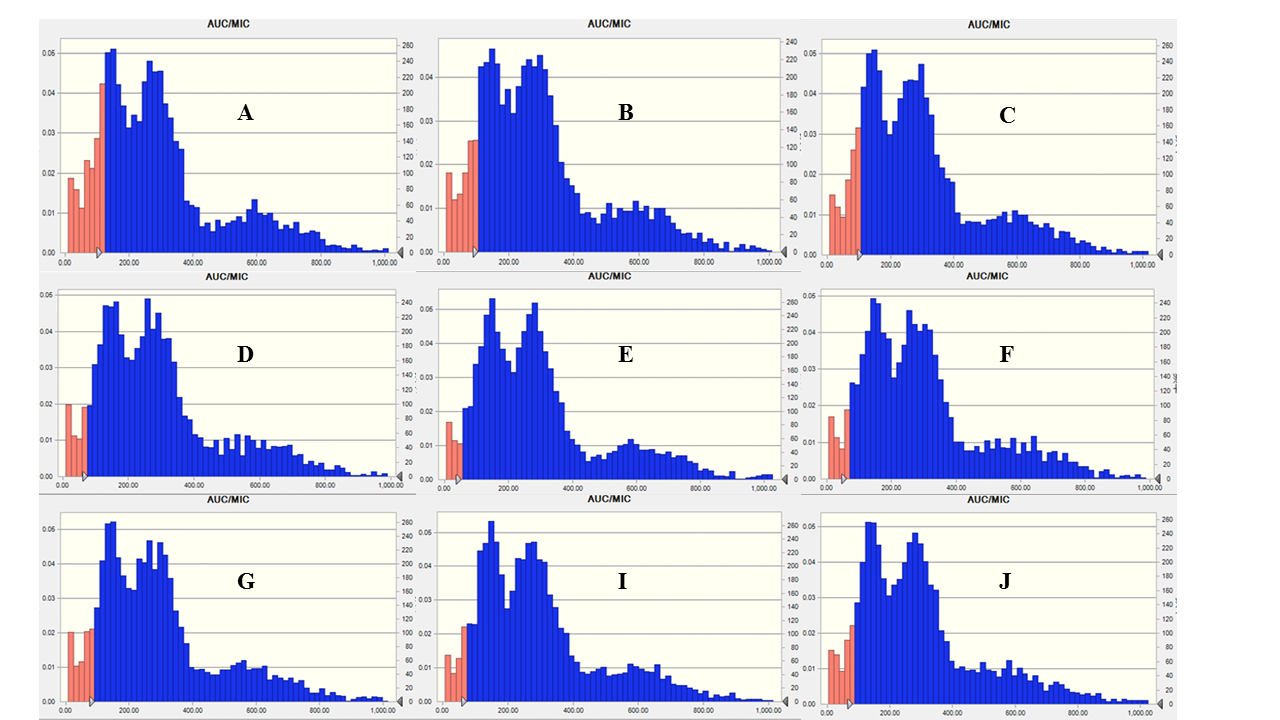

Supplement: Supplementary file 4 [file Image_4.TIF]

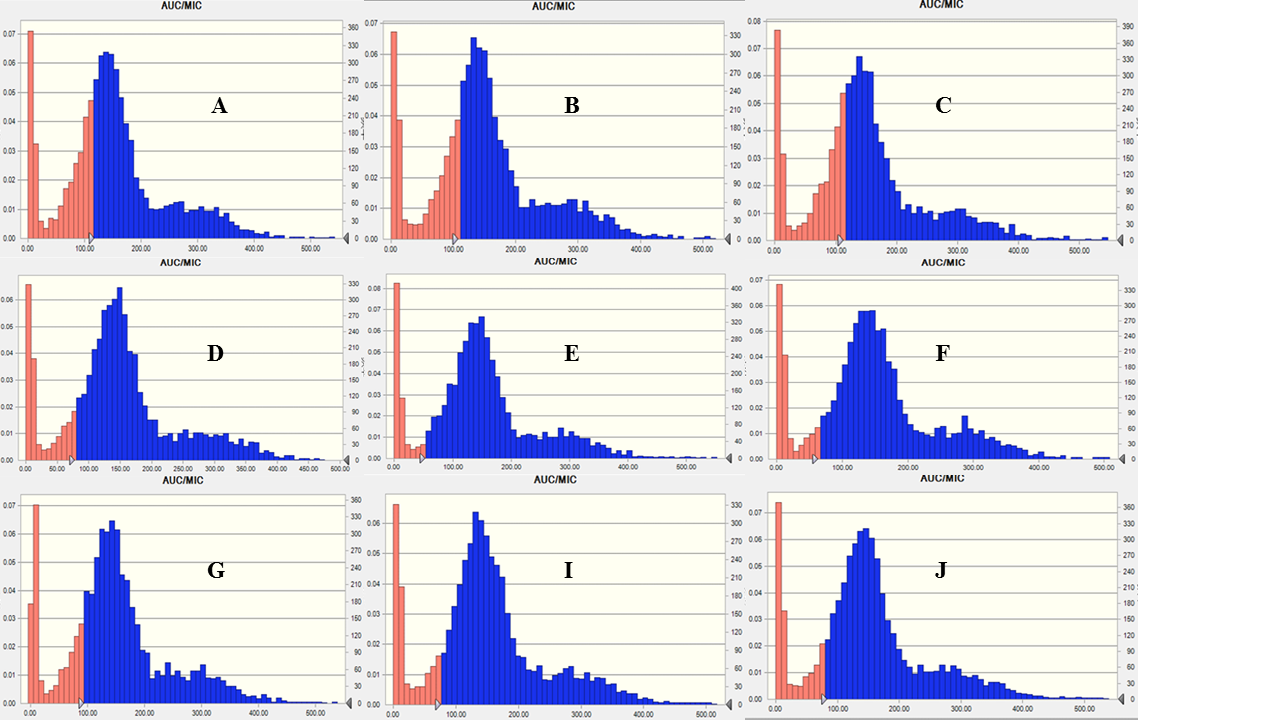

Supplement: Supplementary file 5 [file Image_5.TIF]

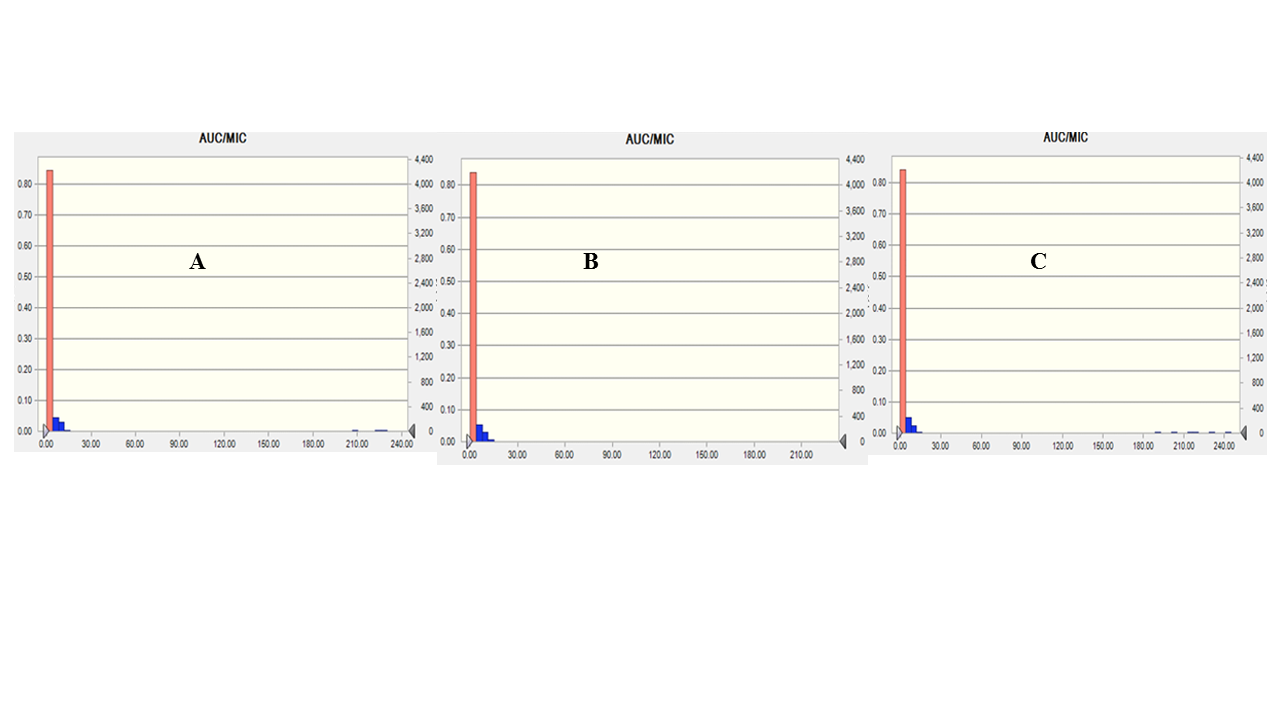

Supplement: Supplementary file 6 [file Image_6.TIF]
