# Supplementary material for: In vivo Pharmacokinetic and Pharmacodynamic (PK/PD) Modeling and Establishment of the PK/PD Cutoff of Florfenicol Against Pasteurella multocida in Ducks
Source: Front Microbiol. 2021 Jan 11;11:616685. doi: 10.3389/fmicb.2020.616685 (PMC7829356; doi:10.3389/fmicb.2020.616685)
Supplement: Supplementary file 7 [file Table_1.DOCX]

**Supplementary Fig.1 The anatomopathological lesions in *P. multocida* infected ducks**

**A, hydropericardium; B, haemorrhages in the heart coronary fat; C, little grey necrosis**

**spots in the liver; D, oedema and haemorrhages in the lung; E, haemorrhages in the intestine**

**Supplementary Fig. 2 The PCR results of isolated strains. A: 0825Y_1_, B: 0901J_1_, C: JY160110**

**Supplementary Fig. 3 The MIC distributions of florfenicol against *P. multocida* in Mainland China, Taiwan China and Globe**

**Supplementary Fig. 4 The Target Attainment Rate (TAR) of florfenicol against *P. multocida* under MIC distribution in Mainland China (using PK/PD surrogate of A, 0825Y_1_-Liver; B, 0825Y_1_-LLung; C, 0825Y_1_-Liver &Lung; D, 0901J_1_-Liver; E, 0901J_1_-Lung; F, 0901J_1_-Liver&Lung; G, 0825Y_1_&0901J_1_-Liver; I, 0825Y_1_&0901J_1_-Lung; J, 0825Y_1_&0901J_1_-Liver&Lung).**

**Supplementary Fig. 5 The Target Attainment Rate (TAR) of florfenicol against *P. multocida* under MIC distribution globely (using PK/PD surrogate of A, 0825Y_1_-Liver; B, 0825Y_1_-LLung; C, 0825Y_1_-Liver &Lung; D, 0901J_1_-Liver; E, 0901J_1_-Lung; F, 0901J_1_-Liver&Lung;G, 0825Y_1_&0901J_1_-Liver; I, 0825Y_1_&0901J_1_-Lung; J, 0825Y_1_&0901J_1_-Liver&Lung).**

**Supplementary Fig. 6 The Target Attainment Rate (TAR) of florfenicol against *P. multocida* under MIC distribution in Taiwan China. (using PK/PD surrogate of A, JY160110 -Liver; B, JY160110-Lung; C, JY160110-Liver &Lung)**
