# Supplementary material for: In vivo Pharmacokinetic and Pharmacodynamic (PK/PD) Modeling and Establishment of the PK/PD Cutoff of Florfenicol Against Pasteurella multocida in Ducks
Source: Front Microbiol. 2021 Jan 11;11:616685. doi: 10.3389/fmicb.2020.616685 (PMC7829356; doi:10.3389/fmicb.2020.616685)
Supplement: Supplementary file 8 [file Table_2.DOCX]

**Supplementary** Table 1 The MIC and MBC values of florfenicol and other antibacterial against twelve P. multocida (μg/mL)

| Strains | MIC in broth | | | | | MIC of florfenicol in serum | MBC of florfenicol | MPC of florfenicol |
| --- | --- | --- | --- | --- | --- | --- | --- | --- |
|  | Cefquinome | Gentamicin | Florfenicol | Tilmicosin | Enrofloxacin |  |  |  |
| C_48-1_ | 0.5 | 4 | 0.25 | 2 | 0.25 | 0.5 | 0.5 | 0.4 |
| JY160110 | 128 | 4 | 32 | 128 | 1 | 32 | 64 | 64 |
| 0612J-L | 1 | 4 | 0.5 | 2 | 2 | 0.5 | 1 | 0.8 |
| 0616J_2_C | 0.5 | 4 | 0.125 | 4 | 0.25 | 0.25 | 0.25 | 0.2 |
| 0616J | 0.5 | 4 | 0.25 | 2 | 0.25 | 0.5 | 0.5 | 0.4 |
| 0621JC | 0.5 | 8 | 0.25 | 2 | 2 | 0.5 | 0.5 | 0.5 |
| 0621J | 0.5 | 4 | 0.25 | 2 | 2 | 0.5 | 0.5 | 0.4 |
| 0725E_1_ | 0.5 | 4 | 0.25 | 2 | 2 | 0.5 | 0.5 | 0.4 |
| 0825Y_1_ | 0.25 | 4 | 0.25 | 1 | 0.25 | 0.5 | 0.5 | 0.4 |
| 0901J_1_ | 1 | 8 | 0.25 | 1 | 2 | 0.5 | 0.5 | 0.4 |
| 0905J_1-1_ | 1 | 8 | 0.25 | 2 | 2 | 0.5 | 0.5 | 0.5 |
| 0908J_3-1_ | 1 | 4 | 0.5 | 2 | 2 | 0.5 | 1 | 0.8 |

**Supplementary** Table 2 The MIC distributions of florfenicol against *P. multocida* derived from the literature

| Region | Separation years | Source of strains | Number of strains | 0.06 | 0.12 | 0.25 | 0.5 | 1 | 2 | 4 | 8 | 16 | 32 | 64 | 128 | 256 | 512 | 1024 | >1024 | MIC_50_ | MIC_90_ |
| --- | --- | --- | --- | --- | --- | --- | --- | --- | --- | --- | --- | --- | --- | --- | --- | --- | --- | --- | --- | --- | --- |
| America and Canada | 2000-2009 | bovine | 3291 | 14 | 29 | 662 | 2019 | 135 | 26 | 39 | 251 | 86 | 28 | 2 |  |  |  |  |  | 0.5 | 8 |
| Canada | 2014-2015 | bovine | 117 |  |  | 12 | 68 | 30 | 5 |  | 2 |  |  |  |  |  |  |  |  | 0.5 | 1 |
| Australia | 2002-2013 | swine | 51 |  | 1 |  | 35 | 13 |  | 1 |  |  |  |  | 1 |  |  |  |  | 0.5 | 1 |
| Mainland | 2003-2007 | swine | 233 | 8 | 48 | 107 | 54 | 9 | 3 | 4 |  |  |  |  |  |  |  |  |  | 0.25 | 0.5 |
| Europe | 2002-2006 | swine and bovine | 231 |  | 3 | 81 | 145 | 1 |  |  |  | 1 |  |  |  |  |  |  |  | 0.5 | 0.5 |
| Europe | 2009-2012 | swine and bovine | 134 |  |  | 17 | 116 | 1 |  |  |  |  |  |  |  |  |  |  |  | 0.5 | 0.5 |
| Spain | 1987-1988 | swine | 63 |  |  | 15 | 47 |  | 1 |  |  |  |  |  |  |  |  |  |  | 0.5 | 0.5 |
|  | 2003-2004 | swine | 132 |  |  | 26 | 92 | 14 |  |  |  |  |  |  |  |  |  |  |  | 0.5 | 1 |
| Taiwan | 2013-2015 | swine | 62 |  |  | 5 |  |  |  |  | 3 | 3 | 14 | 18 | 11 | 2 |  | 1 | 5 | 64 | 256 |
| Globe | 1987-2015 | swine and bovine | 4314 | 22 | 81 | 925 | 2576 | 203 | 35 | 44 | 256 | 90 | 42 | 20 | 12 | 2 |  | 1 | 5 | 0.5 | 4 |
